# Einfluss von Komplikationen und Komorbiditäten auf Liegedauer und Kosten bei der operativen Behandlung der proximalen Humerusfraktur

**DOI:** 10.1007/s00104-021-01491-w

**Published:** 2021-09-17

**Authors:** Josef Stolberg-Stolberg, Jeanette Köppe, Robert Rischen, Moritz Freistühler, Andreas Faldum, J. Christoph Katthagen, Michael J. Raschke

**Affiliations:** 1 Klinik für Unfall‑, Hand- und Wiederherstellungschirurgie, Universitätsklinikum Münster, Albert-Schweitzer-Campus 1, Gebäude W1, 48149 Münster, Deutschland; 2 Institut für Biometrie und Klinische Forschung, Westfälische Wilhelms-Universität Münster, Schmeddingstraße 56, 48149 Münster, Deutschland; 3 Klinik für Radiologie, Universitätsklinikum Münster, Albert-Schweitzer-Campus 1, Gebäude A1, 48149 Münster, Deutschland; 4 Geschäftsbereich Medizinisches Management-Medizincontrolling, Universitätsklinikum Münster, Niels-Stensen-Straße 8, 48149 Münster, Deutschland

**Keywords:** Winkelstabile Plattenosteosynthese, Inverse Schulterprothese, Geriatrische Chirurgie, Multivariable Lineare Regression, Kosteneffektivität, Locked plate fixation, Reverse total shoulder arthroplasty, Geriatric surgery, Multivariable linear Regression, Cost-effectiveness

## Abstract

Nach proximaler Humerusfraktur beim alten Patienten stellen die winkelstabile Plattenosteosynthese und die inverse Schulterendoprothese zwei konkurrierende Operationsverfahren dar. Auch wenn erste klinische Studien auf eine funktionelle Überlegenheit der inversen Schulterendoprothese hindeuten, fehlt ein gesundheitsökonomischer Vergleich in der Literatur. Krankenkassendaten von 55.070 Patienten ab einem Alter von 65 Jahren, welche nach proximaler Humerusfraktur mittels inverser Schulterendoprothese oder winkelstabiler Plattenosteosynthese versorgt worden sind, wurden auf Kosten und Liegedauer untersucht. Multivariable lineare Regressionsmodelle wurden zur Beurteilung von Komplikationen und Komorbiditäten gerechnet. Die Liegedauer nach inverser Schulterendoprothese war mit 20,0 (±13,5) Tagen statistisch auffällig länger als nach winkelstabiler Plattenosteosynthese mit 14,6 (±11,4) Tagen (*p* < 0,001). Die Kosten pro Fall unterschieden sich mit 11.165,70 (±5884,36) EUR für die inverser Prothese und 7030,11 (±5532,02) EUR für die Plattenosteosynthese deutlich (*p* < 0,001). Statistisch auffällige Kostensteigerungen durch Komplikationen und Komorbiditäten unterstreichen den Bedarf an spezialisierten geriatrischen Traumazentren.

## Abstract (en)

After a proximal humeral fracture in older patients, locked plate fixation and reverse total shoulder arthroplasty are two competing surgical procedures. Even if recent clinical studies indicate a functional superiority of reverse shoulder arthroplasty over locked plate fixation, health-economic comparative data are lacking in the literature. Health claims data of 55,070 patients aged 65 years or older who were treated with reverse total shoulder arthroplasty or locked plate fixation after proximal humeral fractures, were analyzed regarding length of hospital stay and costs. Multivariable linear regression models were used to analyze the influence of comorbidities and complications on the length of hospital stay and costs. The length of hospital stay after reverse total shoulder arthroplasty with 20.0 days (±13.5 days) was statistically noticeable longer compared to locked plate fixation with 14.6 days (±11.4 days, *p* < 0.001). The costs per case showed a clear difference with 11,165.70€ (±5884.36€) for reverse total shoulder arthroplasty and 7030.11€ (±5532.02€) for locked plate fixation (*p* < 0.001). Statistically noticeable cost increases due to comorbidities and complications underline the urgent need for specialized geriatric trauma centers.

## Hintergrund

Die proximale Humerusfraktur ist mit einer Inzidenz von 82/100.000 Personenjahren und einem Anteil von 5 % aller Frakturen fester Bestandteil des unfallchirurgischen Alltags [4, 19]. Als Indikatorfraktur für Osteoporose ist sie die dritthäufigste Fraktur des alten Patienten [9, 13, 20, 24]. Prognosen sagen einen dramatischen Anstieg des Bevölkerungsanteil von über 65 Jahre auf bis zu 20 % der Gesamtbevölkerung im Jahr 2050 voraus, sodass mit einem entsprechenden Mehraufkommen dieser Frakturentität zu rechnen ist [5, 15, 29].

Die am häufigsten durchgeführte operative Therapie ist die winkelstabile Plattenosteosynthese [11, 15]. Trotz kontinuierlicher Weiterentwicklung des Operationsverfahrens und der Implantate ist insbesondere am osteoporotischen Knochen mit einer hohen Komplikationsrate zu rechnen [21, 32]. Als konkurrierendes Verfahren zeigen aktuelle Zahlen, dass sich die inverse Schulterendoprothese als zuverlässiges und beliebtes Implantat in Deutschland etabliert hat [12]. Obwohl erste klinische Studien eine Überlegenheit der inversen Schulterprothese gegenüber der winkelstabilen Plattenosteosynthese nahelegen, ist ein endgültiger wissenschaftlicher Nachweis noch ausstehend [6, 10, 16]. Eine aktuelle Risikoanalyse belegt zudem, dass die inverse Schulterprothese mit erhöhten Komplikationsraten während des primären stationären Aufenthaltes assoziiert ist [18]. Insbesondere unter dem Aspekt der deutlich höheren Kosten^1^ der inversen Schulterendoprothese, gilt es, den aktuellen Trend und die Zunahme der endoprothetischen Implantationsraten kritisch zu hinterfragen.

Ziel dieser Arbeit ist es, Kosten und stationäre Liegedauer zwischen inverser Schulterendoprothese und winkelstabiler Plattenosteosynthese zu vergleichen sowie den Einfluss von Komorbiditäten und Komplikationen zu untersuchen. Die Hypothese lautet daher, dass Komorbiditäten und Komplikationen nach inverser Schulterendoprothetik sowie winkelstabiler Plattenosteosynthese während des stationären Aufenthaltes nicht nur zu einer verlängerten Liegedauer, sondern auch zu einem Kostenanstieg führen.

## Material und Methode

### Daten und Patientenkohorte

Die vorliegende Studie wurde durch die Ethikkommission der Ärztekammer Westfalen-Lippe genehmigt (Aktenzeichen 2020-160-f-S). Die bundesweiten Versichertendaten wurden freundlicherweise durch den größten deutschen gesetzlichen Krankenversicherer, der Allgemeine Ortskrankenkasse (AOK), mit ca. 26,5 Mio. Versicherungsnehmern zur Verfügung gestellt. Durch die einheitlichen gesetzlichen Vorgaben zur Codierung der Diagnosen über ICD (International Statistical Classification of Diseases, German Modification) und Prozeduren über den Operationen- und Prozedurenschlüssel (OPS) zeichnen sich die genutzten Abrechnungsdaten insbesondere durch ihre Vollständigkeit aus.

Für den Analysezeitraum von Januar 2010 bis inkl. September 2018 wurden alle Patienten ab einem Alter von 65 Jahren zum Zeitpunkt der Hospitalisierung, die mit einer winkelstabilen Plattenosteosynthese (OPS: 5‑794.k1 oder 5‑794.21) oder inversen Schulterprothese (OPS: 5‑824.21) und der Diagnose einer proximalen Humerusfraktur (ICD: S42.2) codiert wurden, eingeschlossen. Ausgeschlossen wurden alle Patienten mit unvollständigen Datensätzen (*n* = 357), nach Polytrauma (*n* = 220), im Vorfeld stattgehabter Versorgung mit winkelstabiler Plattenosteosynthese oder inverser Schulterprothese (*n* = 35) oder Knochentumoren (*n* = 298). Vorerkrankungen wurden anhand der stationär und ambulant codierten Diagnosen und Prozeduren der vergangenen 24 Monate erfasst. Der Charlson Comorbidty Index (CCI) wurde entsprechen der Definition von Quan et al. berechnet und dem German ICD-10 angepasst [3, 23, 30].

Als primäre Fragestellung wurde untersucht, inwieweit sich – bei Vorhandensein unterschiedlicher Komplikationen während der Hospitalisierung – die Fallkosten und die Liegedauer zwischen den Patienten, die mit einer inversen Schulterprothese behandelt wurden, von denen mit einer winkelstabilen Plattenosteosynthese unterscheiden. Untersucht wurden hierbei:

- chirurgische Komplikationen (Infektion, Infektion mit antibiotikaresistenten Keimen, Bursitis, Kompartmentsyndrom, postoperatives Hämatom, Impingement, Implantatversagen, periprothetische oder periimplantäre Fraktur, Nervenverletzung, Gefäßverletzung, Revisionseingriff);
- implantatassoziierte Komplikationen (Impingement, Bursitis, mechanische Komplikation, Materialversagen, Implantatlockerung, periprothetische oder periimplantäre Fraktur);
- nicht-implantatassoziierte Komplikationen (Infektion, Infektion mit antibiotikaresistenten Keimen, postoperatives Hämatom);
- allgemeine Komplikationen (Herzinfarkt, Lungenembolie, Schlaganfall, tiefe Venenthrombose, akutes Nierenversagen, akutes Leberversagen, akutes Lungenversagen („acute respiratory distress syndrome“, ARDS), Sepsis, Delir, Intensivpflichtigkeit, Reanimation und Schock);
- thromboembolische Ereignisse (Lungenembolie, ischämischer Schlaganfall, tiefe Venenthrombose).

Weitere Details zur Definition der einzelnen Punkte sind in Köppe et al. zu finden [18]. Als sekundäre Analysen wurde untersucht, wie die einzelnen Komplikationen, unter Berücksichtigung des individuellen Patientenprofils (CCI, Alter, Geschlecht), die Liegedauer und die Fallkosten erhöhen. Zudem wurde der Einfluss der einzelnen Komorbiditäten auf die Liegedauer und die Fallkosten untersucht.

### Statistische Analyse

Zur Überprüfung der primären Fragestellungen wurden die Unterschiede zwischen „reverse total shoulder arthroplasty“ (RTSA, inverse Schulterprothese) und „locked plate fixation“ (LPF, winkelstabile Plattenosteosynthese) bei Patienten, bei denen eine entsprechende Komplikation auftrat, mittels zweiseitigem Mann-Whitney-U-Test überprüft. Zusätzlich wurden multivariable lineare Regressionsmodelle für die Liegedauer und die Kosten berechnet, wobei Alter, Geschlecht, CCI, Jahr der Hospitalisierung und die oben genannten Komplikationen berücksichtigt wurden. Hierdurch kann sowohl der Unterschied zwischen beiden Behandlungsgruppen unter Berücksichtigung der genannten Punkte evaluiert werden, als auch der Einfluss der betrachteten Komplikationen auf die Fallkosten bzw. die Liegedauer analysiert werden. Zusätzlich wurden weitere Modelle berechnet, indem, neben Alter, Geschlecht, Behandlungsgruppe und Jahr der Hospitalisierung, das individuelle Komorbiditätsprofil des Patienten berücksichtigt wurde.

Es handelt sich bei der vorliegenden Studie um eine rein explorative Analyse; eine Adjustierung der *p*-Werte zur Berücksichtigung des multiplen Testproblems erfolgte nicht. Alle *p*-Werte < 5 % werden als statistisch auffällig im Sinne der Hypothesengenerierung interpretiert. Zur Analyse wurde SAS Software (V9.4, *SAS *Institute Inc., Cary, NC, USA) und R Version 3.6.0 (2019-04-26, R Foundation for Statistical Computing, Wien, Österreich) verwendet.

## Ergebnisse

### Demografische Aspekte, Kosten und Liegedauer

Im Studienzeitraum vom 01.01.2010 bis 30.09.2018 wurden insgesamt 55.070 Patienten mit einem medianen (Q1, Q3) Alter von 79 (74, 84) Jahren und einem Anteil von 84,4 % Frauen eingeschlossen. Der überwiegende Teil (41.216, 74,8 %) der Patienten wurde mit einer winkelstabilen Plattenosteosynthese versorgt, 13.854 (25,2 %) der Patienten erhielten eine inverse Schulterprothese (Tab. 1).

Wie in einer früheren Arbeit bereits im Detail erläutert, waren die Patienten der RTSA-Gruppe älter und wiesen eine höhere Prävalenz von Begleiterkrankungen auf [18]. Zudem war die Liegedauer der Patienten mit inverser Schulterprothetik im Mittel deutlich länger als bei den Patienten mit LPF (RTSA: Mittelwert [± Standardabweichung, STD] 20,0 [±13,5] Tage vs. LPF 14,6 [±11,4]; *p* < 0,001). Auch die mittleren (± STD) Kosten pro Fall war bei Patienten mit inverser Schulterprothese mit 11.165,70 (±5884,36) EUR wesentlich höher als bei Patienten mit winkelstabiler Plattenosteosynthese mit 7030,11 (±5532,02) EUR (*p* < 0,001). Im Studienzeitraum stiegen die mittleren (± STD) Kosten pro Fall in der RTSA-Gruppe von 9737,14 (±3036,44) EUR im Jahr 2010 auf 11.791,71 (±4246,15) EUR im Jahr 2018, während die mittlere Liegedauer abnahm (21,4 [±12,8] Tage im Jahr 2010 auf 18,9 [±11,9] Tage im Jahr 2018). Diese Trends wurden auch in der LPF-Gruppe beobachtet (Tab. 2).

**Table 1:** 

	Gesamtkohorte *n* = 55.070 (100,0 %)	LPF *n* = 41.216 (74,8 %)	RTSA *n* = 13.854 (25,2 %)	*p*-Wert
Anteil Frauen – *n* (%)	46.488 (84,4 %)	34.476 (83,7 %)	12.012 (86,7 %)	< 0,001
Medianes Alter (Q1, Q3) – Jahre	79 (74, 84)	78 (73, 84)	81 (76, 85)	< 0,001
Medianer CCI (Q1, Q3)	3 (1, 4)	2 (1, 4)	3 (1, 4)	< 0,001
Mittlere Fallkosten (± STD) – EUR	8070,50 (±5902,11)	7030,11 (±5532,02)	11.165,70 (±5884,36)	< 0,001
Mittlere Liegedauer (± STD) – Tage	16,0 (±12,2)	14,6 (±11,4)	20,0 (±13,5)	< 0,001

*CCI* Charlson Comorbidity Index, *LPF* „locked plate fixation“, *RTSA* „reverse total shoulder arthroplasty“, *STD* Standardabweichung

**Table 2:** 

	2010	2011	2012	2013	2014	2015	2016	2017	2018
*Mittlerer Kosten pro Fall – EUR* *±* *STD*									
LPF	6015,92 ±4241,44	6389,70 ±4489,04	6557,13 ±4490,98	6807,81 ±5588,83	7319,34 ±6075,93	7463,70 ±7285,68	7607,96 ±6025,08	7710,94 ±5541,24	8028,92 ±5427,86
RTSA	9737,14 ±3036,44	9813,57 ±3737,06	10.128,39 ±3907,57	10.381,77 ±3506,02	11.080,42 ±6649,99	11.256,53 ±6798,99	11.346,00 ±7271,84	11.874,71 ±6758,47	11.791,71 ±246,15
Gesamt	6325,55 ±4279,60	6839,94 ±4546,13	7157,04 ±4596,29	7540,90 ±5424,91	8290,60 ±6442,56	8537,73 ±7352,07	8853,79 ±6702,62	9236,03 ±6340,98	9578,29 ±308,35
*Mittlere Liegedauer – Tage* *±* *STD*									
LPF	15,3 ± 10,8	15,6 ± 11,9	14,9 ± 10,8	14,6 ± 11,4	14,6 ± 11,4	14,4 ± 12,0	14,0 ± 11,0	14,0 ± 12,1	13,8 ± 11,4
RTSA	21,4 ± 12,8	21,1 ± 13,3	20,6 ± 12,8	20,3 ± 12,6	20,5 ± 13,2	20,2 ± 13,7	19,9 ± 13,5	19,5 ± 15,5	18,9 ± 11,9
Gesamt	15,8 ± 11,1	16,3 ± 12,3	15,8 ± 11,4	15,8 ± 11,9	16,1 ± 12,2	16,0 ± 12,8	15,9 ± 12,2	16,0 ± 13,7	15,9 ± 11,8

*LPF* „locked plate fixation“, *RTSA* „reverse total shoulder arthroplasty“, *STD* Standardabweichung

### Einfluss von Komplikationen auf Erlös und Liegedauer

**Figure 1:**
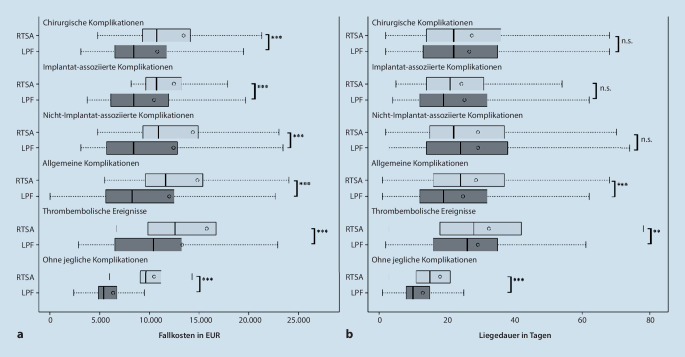

Wie in Tab. 3 dargestellt, erhöhten Komplikationen während des Krankenhausaufenthalts die Kosten in beiden Gruppen etwa gleich stark. Darüber hinaus war die Liegedauer durch Komplikationen in beiden Gruppen stark erhöht (Mittelwert [±STD] ohne Komplikationen: 14,1 ± [9,5] Tage vs. mit Komplikationen: 25,3 ± [18,3] Tage, *p* < 0,001; Tab. 3). In Abb. 1 ist der Einfluss unterschiedlicher Komplikationen auf den Erlös und die Liegedauer in Abhängigkeit der Behandlungsgruppe dargestellt. Es ist zu erkennen, dass durch alle dort gezeigten Komplikationen die Kosten in beiden Behandlungsgruppen enorm anstiegen, wobei im Mittel in allen dort gezeigten Punkten die inverse Prothese mit höheren Kosten verbunden war (alle *p*-Werte *p* < 0,001). Auch die Liegedauer wurde bei allen untersuchten Komplikationen in beiden Behandlungsgruppen deutlich erhöht, wobei der Unterschied zwischen Prothese und winkelstabiler Platte bei den chirurgischen Komplikationen statistisch nicht auffällig war (*p* > 5 %).

**Table 3:** 

	Jegliche Komplikation während der Hospitalisierung		*p*-Wert
	Nein	Ja	
*Mittlerer Kosten pro Fall – EUR* *±* *STD*			
LPF	6355,44 ± 2830,56	10.832,55 ± 11.871,04	< 0,001
RTSA	10.460,87 ± 2842,80	13.778,79 ± 11.153,48	< 0,001
Gesamt	7331,01 ± 3328,95	11.779,76 ± 11.725,59	< 0,001
*Mittlere Liegedauer – Tage* *±* *STD*			
LPF	12,9 ± 8,8	24,3 ± 17,9	< 0,001
RTSA	18,0 ± 10,8	27,4 ± 18,9	< 0,001
Gesamt	14,1 ± 9,5	25,3 ± 18,3	< 0,001

*LPF* „locked plate fixation“, *RTSA* „reverse total shoulder arthroplasty“, *STD* Standardabweichung

In Tab. 4 ist, unter gleichzeitiger Berücksichtigung von Alter, Geschlecht, CCI und Behandlungsgruppe des Patienten, der Einfluss unterschiedlicher Komplikationen auf die Kosten und die Liegedauer dargestellt. Die Notwendigkeit einer weiteren Operation an der Schulter (β = 12,6 Tage, 95 % Konfidenzintervall [KI]: 12,1–13,0 Tage), Sepsis (β = 13,6 Tage, 95 %-KI: 12,4–14,9 Tage), Hirnblutung (β = 14,2 Tage, 95 %-KI: 12,0–16,5 Tage) und thrombembolische Ereignisse (β = 10,0 Tage, 95 %-KI: 9,2–10,9) steigerten die Liegedauer im Mittel am meisten (alle *p*-Werte < 0,001). ARDS (β = 22.916,14 EUR), Sepsis (β = 18.452,66 EUR) und Hirnblutung (β = 13.290,02 EUR) sind zudem im Mittel mit einer starken Erhöhung der Fallkosten assoziiert (beide *p*-Werte *p* < 0,001). Eine weitere Operation während der Hospitalisierung war, trotz deutlich erhöhter Liegedauer, nur mit einer mittleren Erhöhung der Kosten um β = 2711,81 EUR assoziiert (*p* < 0,001).

**Table 4:** 

	Liegedauer in Tagen			Kosten in EUR		
	β	95 %-KI	*p*-Wert	β	95 %-KI	*p*-Wert
*RTSA vs. LPF*	4,16	3,94–4,37	< 0,001	3224,70	3125,20–3324,19	< 0,001
Geschlecht (Männer vs. Frauen)	−0,01	−0,25–0,24	0,967	277,17	161,94–392,41	< 0,001
Alter pro Jahr	0,15	0,14–0,17	< 0,001	6,96	0,94–12,98	0,023
CCI pro Scorepunkt	0,40	0,36–0,44	< 0,001	84,67	66,48–102,85	< 0,001
Indexjahr	−0,21	−0,242–−0,17	< 0,001	270,81	254,14–287,48	< 0,001
*Komplikationen im Indexfall*						
Weitere Operationen	12,55	12,09–13,01	< 0,001	2711,81	2498,12–2925,50	< 0,001
Akutes Nierenversagen	4,53	3,91–5,14	< 0,001	2201,49	1916,35–2486,63	< 0,001
ARDS	8,11	3,80–12,42	< 0,001	22.916,14	20.917,72–24.914,56	< 0,001
Akutes Leberversagen	−3,11	−5,64–−0,57	0,016	−2691,75	−3868,66–−1514,83	< 0,001
Sepsis	13,62	12,39–14,86	< 0,001	18.452,66	17.880,28–19.025,03	< 0,001
Delir	4,11	3,68–4,54	< 0,001	1330,79	1130,90–1530,69	< 0,001
Thrombembolisches Ereignis	10,04	9,19–10,88	< 0,001	3644,17	3253,23–4035,10	< 0,001
Akuter Myokardinfarkt	3,11	2,00–4,22	< 0,001	2130,44	1617,20–2643,68	< 0,001
Hämorrhagischer Schlaganfall	14,22	11,97–16,47	< 0,001	13.290,02	12.246,29–14.333,74	< 0,001
Nichtimplantatassoziierte Komplikationen	6,54	5,99–7,10	< 0,001	2455,79	2198,72–2712,86	< 0,001
Implantatassoziierte Komplikationen	2,38	1,34–3,43	< 0,001	558,86	74,11–1043,62	0,024
Behandlung Intensivstation	4,75	4,20–5,31	< 0,001	3572,07	3313,67–3830,47	< 0,001
Bluttransfusion	5,38	5,13–5,63	< 0,001	2142,51	2026,59–2258,43	< 0,001
Achsenabschnitt β_0_	413,4	341,1–485,8	< 0,001	−539.996	−573.568–−506.423	< 0,001

*ARDS* „acute respiratory distress syndrome“, *CCI* Charlson Comorbidity Index, *LPF* „locked plate fixation“, *RTSA* „reverse total shoulder arthroplasty“

Darüber hinaus ist anzumerken, dass insbesondere bei komplikationslosen Verläufen sowohl Kosten als auch Liegedauer bei der inversen Prothese höher waren als bei Patienten mit winkelstabiler Plattenosteosynthese (beide *p*-Werte *p* < 0,001; siehe Abb. 1 und Tab. 6 im Anhang). Nach Adjustierung auf Alter, Geschlecht, Begleiterkrankungen (über CCI) bestätigte sich dieser Effekt. Bei einem komplikationslosen Verlauf, gleichem Alter, Geschlecht und CCI war die inverse Prothese im Mittel (95 %-KI) mit β = 3224,70 EUR (3125,20–3324,19 EUR) höherem Erlös und einer im Mittel um β = 4,2 Tage (3,9–4,4 Tage) erhöhten Liegedauer assoziiert (beide *p* < 0,001; siehe Tab. 4).

### Einfluss der Komorbiditäten auf Kosten und Liegedauer

Unter Berücksichtigung des individuellen Risikoprofils zeigt sich, dass Alkoholmissbrauch (β = 2,8 Tage, 95 %-KI: 2,3–3,2 Tage), Vorhofflimmern (β = 2,3 Tage, 95 %-KI: 2,0–2,6 Tage), chronisches Nierenversagen (β = 2,1 Tage, 95 %-KI: 1,9–2,4 Tage), Parkinson (β = 2,0 Tage, 95 %-KI: 1,5–2,4 Tage) und Herzinsuffizienz (β = 1,9 Tage, 95 %-KI: 1,7–2,2 Tage) jeweils mit einer deutlichen Erhöhung der Liegedauer (alle *p*-Werte *p* < 0,001) und ebenfalls mit einer entsprechenden Erhöhung der mittleren Fallkosten assoziiert sind (alle *p*-Werte *p* < 0,001; Tab. 5). Auch eine codierte Demenzerkrankung zum Zeitpunkt der Operation ist mit einer mittleren Kostensteigerung von β = 682,12 EUR (95 %-KI: 477,28–886,95 EUR; *p* < 0,001) assoziiert.

**Table 5:** 

	Liegedauer in Tagen			Kosten in EUR		
	β	95 %-KI	*p*-Wert	β	95 %-KI	*p*-Wert
RTSA vs. LPF	4,85	4,62–5,08	< 0,001	3620,72	3509,70–3731,74	< 0,001
Geschlecht (Männer vs. Frauen)	0,93	0,64–1,21	< 0,001	725,84	590,19–861,50	< 0,001
Alter pro Jahr	0,20	0,18–0,22	< 0,001	28,16	20,87–35,453	< 0,001
Indexjahr	−0,29	−0,33–−0,25	< 0,001	238,73	220,05–257,42	< 0,001
Osteoporose	1,37	1,16–1,58	< 0,001	392,37	292,55–492,19	< 0,001
Krebsdiagnose	0,04	−0,19–0,28	0,712	1,65	−110,28–113,58	0,977
Diabetes Mellitus	0,39	0,18–0,60	< 0,001	156,18	56,59–255,78	0,002
Demenz	1,05	0,62–1,48	< 0,001	682,12	477,28–886,95	< 0,001
Polyarthritis, chronisch	0,01	−0,40–0,42	0,962	166,19	−27,72–360,09	0,093
Übergewicht	0,41	0,18–0,64	< 0,001	−146,61	−255,49–−37,72	0,008
Nikotinabusus	0,89	0,46–1,32	< 0,001	386,91	180,60–593,21	< 0,001
Parkinson	1,95	1,46–2,44	< 0,001	534,48	299,56–769,40	< 0,001
Ruptur Rotatorenmanschette	−0,82	−1,19–−0,44	< 0,001	−160,84	−340,74–19,07	0,080
Alkoholabusus	2,75	2,30–3,20	< 0,001	657,49	443,14–871,83	< 0,001
Vorangegangener Schlaganfall	0,15	−0,08–0,38	0,191	124,90	14,83–234,97	0,026
Omarthrose	−1,37	−2,02–−0,72	< 0,001	−457,66	−767,50–−147,82	0,004
„Frozen shoulder“	−0,39	−0,86–0,087	0,110	−264,45	−490,30–−38,60	0,022
Vorangegangene Operationen an der Schulter	−1,28	−2,77–0,22	0,095	−291,49	−1006,67–423,69	0,424
Vorhofflimmern	2,30	2,04–2,56	< 0,001	819,90	695,76–944,05	< 0,001
Herzinsuffizienz	1,93	1,70–2,17	< 0,001	631,56	519,23–743,89	< 0,001
Koronare Herzkrankheit	0,19	−0,04–0,42	0,101	35,61	−72,98–144,20	0,520
Hypertonus	0,15	−0,16–0,47	0,339	−77,08	−227,61–73,45	0,316
Atherosklerose	0,39	0,12–0,65	0,004	156,83	29,93–283,73	0,015
Chronisches Nierenversagen	2,13	1,90–2,36	< 0,001	551,32	439,48–663,15	< 0,001
Achsenabschnitt β_0_	578,5	499,7–657,4	< 0,001	−476773	−514409–−439137	< 0,001

*CCI* Charlson Comorbidity Index, *LPF* „locked plate fixation“, *RTSA* „reverse Total Shoulder arthroplasty“

## Diskussion

Die wichtigsten Ergebnisse dieser Studie belegen, dass die inverse Schulterprothese nach proximaler Humerusfraktur im Vergleich zur winkelstabilen Plattenosteosynthese nicht nur bei einem komplikationsfreien Verlauf höhere Kosten generiert, sondern auch die komplikativen Verläufe hoch signifikant teurer abgerechnet werden. Die stationäre Liegedauer gleicht sich zwischen beiden Verfahren nach einer Komplikation an. Der Einfluss von Alter, Geschlecht, CCI, spezifischen Nebenerkrankungen und Komplikationen auf die Liegedauer und Fallkosten konnte quantifiziert werden.

Das G‑DRG- und OPS-System wurden Anfang des Jahrtausends mit dem Ziel der Einführung eines leistungsorientierten und pauschalisierten Vergütungssystems initiiert. Seitdem erfolgte ein Rückgang der stationären Verweildauer um 15 %. Zeitgleich konnte aber auch eine Rückkehr zum Anstieg der stationären Fälle verzeichnet werden und somit ist dieses System durchaus kritikwürdig [2]. In Bezugnahme auf die proximale Humerusfraktur beim Patienten über 65 Jahre zeigen unsere Daten während des Indexzeitraums einen mäßigen Anstieg der Gesamtoperationszahlen mit 5985 Fällen im Jahr 2010 und 6992 Fällen im Jahr 2017, was in Teilen durch den demografischen Wandel zu erklären ist. Gleichzeitig stieg aber der Anteil der inversen Schulterprothesen von 8 % auf 41 % [18]. Gründe hierfür können die mit zunehmendem Alter einhergehende Morbidität, aber auch ein verbessertes Prothesendesign und operative Erfahrung in der Breite sein [26, 27]. Die hohe Zufriedenheit der behandelnden Unfallchirurgen deckt sich auch mit den Ergebnissen der Delphi-Studie, welche in einem randomisiert-kontrollierten Multicenterstudiendesign eine bessere Schulterfunktionalität gemessen am Constant Score nach inverser Schulterprothese im direkten Vergleich zur winkelstabilen Plattenosteosynthese nach AO(Arbeitsgemeinschaft für Osteosynthesefragen)-Typ-B2- und -C2-Frakturen in einem 2‑Jahres-Follow-up nachweisen konnte [6]. Auch wenn andere Studien geringerer Qualität teils abweichende Ergebnisse präsentieren, ist hiermit nun die rationale Grundlage für die Frakturendoprothetik beim alten Patienten an der Schulter gelegt worden und somit besteht ein dringender Forschungsbedarf nicht nur zu den klinischen, sondern insbesondere auch zu den ökonomischen Aspekten [10, 16].

Die Dauer des stationären Aufenthaltes nach winkelstabiler Plattenosteosynthese oder inverser Schulterendoprothese variiert international zwischen einem Tag und mehreren Wochen [33]. Insbesondere im direkten Vergleich zum angloamerikanischen System wirft die vergleichsweise lange Liegedauer in unserer Studie die Frage nach einer poststationären Versorgungslücke des alten Patienten auf [33]. Im Gegensatz zu elektiven Operationen ist nach proximaler Humerusfraktur eine präoperative Planung der nachstationären Versorgung zwar deutlich erschwert, dennoch kann eine zeitgerechte Evaluation des Rehabilitationsbedarfs und die Einleitung der entsprechenden organisatorischen Schritte durch den Sozialdienst die Liegedauer verkürzen [8]. Modellprojekte wie die multimodale Delirprävention zeigten zudem insbesondere beim geriatrischen Patienten Verbesserungen in der Versorgungsqualität und damit in einer Verkürzung der Liegedauer [31, 35]. Des Weiteren können neben der stationären Rehabilitation abgestufte geriatriespezifische Versorgungsstrukturen, wie z. B. die geriatrische Tagesklinik, die ambulante geriatrische Rehabilitation und die mobile geriatrische Rehabilitation, helfen, die Versorgungslücke zu schließen [1]. In Bezugnahme auf die operative Indikationsstellung verlängern bekannterweise Diabetes mellitus, chronische Arthritis und Schmerzen, Komplikationen, Bluttransfusionen und fehlende häuslich-soziale Unterstützung die Länge des stationären Aufenthaltes [14, 22]. Unsere hier präsentierten Daten vervollständigen nun dieses Wissen um eine Vielzahl an Komorbiditäten sowie Komplikationen und können zusammen mit den durch Köppe et al. publizierten Angaben verwendet werden, um präoperativ das Komplikationsrisiko basierend auf Geschlecht, Alter und Vorerkrankungen patientenindividuell zu berechnen und Liegedauern adaptiert auf Komorbiditäten und Komplikationen zu evaluieren [18]. So ist in Zukunft eine differenziertere Beurteilung der individuell geriatrischen Behandlungsverläufe von und gegenüber den Krankenkassen möglich.

Als Grundlage zur Bewertung der Kosteneffektivität der konkurrierenden operativen Verfahren inverse Schulterendoprothese vs. winkelstabile Plattenosteosynthese am proximalen Humerus konnten in dieser Studie die jeweiligen Kosten ausgewertet werden. Limitierend ist hier hervorzuheben, dass die durch eine medizinische Behandlung anfallenden Kosten teils erheblich von den erzielten Erlösen abweichen [34]. So können die Kosten in der Unfallchirurgie aufgrund des hohen Fixkostenanteils teils erheblich von der Kapazitätsauslastung des Krankenhauses abhängen [25]. Außerdem ist das Fallpauschalenentgeltsystem insbesondere beim alten Patienten kritisch zu bewerten [28]. In Bezugnahme auf die teils erheblichen zusätzlichen Kosten, die bei einem komplikativen Verlauf anfallen, verweisen die Autoren auf Studien, die signifikant geringere Komplikationsraten in Kliniken mit interdisziplinär-geriatrischer Kompetenz und Alterstraumazentren im Vergleich zu nichtgeriatriespezialisierten Krankenhäusern nachweisen konnten: Zum Beispiel konnten Knobbe et al. zeigen, dass die interdisziplinäre Kooperation inklusive einer geriatrischen Kompetenz im Visitenmodell zu einem Rückgang der Letalität von 9 % auf 2 %, kardiopulmonaler Komplikationen von 39 % auf 28 %, von Myokardinfarkt von 6 % auf 0 %, Elektrolytstörungen von 34 % auf 19 % und Exikose von 6 % auf 0 % führte [17]. Konsequenterweise muss hieraus geschlussfolgert werden, dass bei dem zu erwartenden drastischen Fallzahlanstieg geriatrischer Patienten in der Unfallchirurgie die flächendenkende Einrichtung von Alterstraumazentren und geriatrischer Versorgungsstrukturen unabdingbar ist, um eine Wirtschaftlichkeit der Behandlung bei gleichzeitig hoher Qualität auch weiterhin zu gewährleisten.

Zukünftige Forschung muss des Weiteren untersuchen, in welchem Ausmaß sich die Behandlungsverläufe der inversen Schulterprothese und winkelstabilen Plattenosteosythese in der Langzeitbeobachtung unterscheiden. Neben den medizinischen Behandlungskosten müssen zur Kosteneffektivitätsanalyse und gesamtgesellschaftlichen Beurteilung insbesondere die Kosten der Anschlussheilbehandlung, häuslichen Versorgung und möglicherweise der Pflegebedürftigkeit berücksichtigt werden.

### Limitationen der Studie

Zu den Limitationen dieser Studie gehört, dass die verwendeten Daten vornehmlich für finanzielle und betriebswirtschaftliche Zwecke erhoben wurden. Es können keine Aussagen zur individuellen Indikationsstellung der operativen Therapiemodalität oder medizinischen Entscheidungen getroffen werden. Daher kann möglicherweise der Auswahleffekt die Daten verzerren.

## Schlussbetrachtung

Zusammenfassend verursachen Patienten, die mit einer inversen Schulterendoprothese nach proximaler Humerusfraktur versorgt worden sind – mit oder ohne Komplikationen –, signifikant mehr direkte Behandlungskosten. Die Liegedauer beider Verfahren (inverse Schulterendoprothese und winkelstabile Plattenosteosynthese) unterscheiden sich im Falle eines komplikationslosen Verlaufs sowie nach allgemeinen und thrombembolischen Ereignissen zugunsten der winkelstabilen Plattenosteosynthese.

Fazit dieser Studie ist, dass zur Senkung der Komplikationsraten und damit der Kosten präoperativ eine sorgfältige und individuelle Risikoabschätzung erfolgen sollte und die weitere Etablierung von Alterstraumazentren sowie geriatrischer Versorgungsstrukturen unabdingbar für die zukünftige Wirtschaftlichkeit sind.

## Appendix

**Table 6:**

	LPF		RTSA		
	Median (Q1; Q3)	Mittelwert (± STD)	Median (Q1, Q3)	Mittelwert (± STD)	*p‑Wert*
*A: Fallkosten in EUR*					
Ohne Komplikationen	5392,95 (4890,73; 6720,25)	6355,44 (±2830,56)	9614,22 (9071.08; 11.155,20)	10.460,87 (±2842,80)	< 0,001
Thrombembolisches Ereignis	10.357,12 (6482,90; 11.553,53)	13.275,04 (±18.388,65)	12.534,28 (9797,79; 16.666,83)	15.742,95 (±14.276,18)	< 0,001
Allgemeine Komplikationen	8211,18 (5566,25; 12.439,35)	11.960,28 (±14.558,78)	11.589,08 (9552,68; 15.348.51)	14.824,85 (±13.229,90)	< 0,001
Chirurgische Komplikationen	8369,38 (6499,29; 6776,27)	10.757,70 (±10.849,51)	10.708,15 (9279,86; 14.103,17)	13.429,99 (±11.223,64)	< 0,001
Nichtimplantatassoziierte Komplikationen	8359,84 (5652,12; 12.773,29)	12.377,86 (±15.810,50)	10.872,81 (9296,47; 14.901,59)	14,366,50 (±13.272,44)	< 0,001
Implantatassoziierte Komplikationen	8372,47 (6089,66; 11.868,54)	10.424,03 (±8593,02)	10.676,03 (9603,51; 13.220,61)	12.417,79 (±4994,27)	< 0,001
*B: Liegedauer in Tagen*					
Ohne Komplikationen	10 (8; 15)	12,9 (±8,8)	15 (11; 21)	18,0 (±10,8)	< 0,001
Thrombembolisches Ereignis	26 (16; 35)	29,3 (±21,9)	28 (18; 42)	32,5 (±20,2)	0,010
Allgemeine Komplikationen	19 (12; 32)	24,7 (±19,7)	24 (16; 37)	28,7 (±20,3)	< 0,001
Chirurgische Komplikationen	22 (13; 35)	26,6 (±19,2)	22 (14; 36)	27,3 (±18,5)	0,089
Nichtimplantatassoziierte Komplikationen	24 (14; 38)	29,3 (±22,0)	22 (15; 37)	29,3 (±20,7)	0,486
Implantatassoziierte Komplikationen	19 (12; 32)	25,1 (±20,7)	21 (14; 31)	24,3 (±14,3)	0,189

*LPF* Locked Plate Fixation, *RTSA* Reverse total shoulder arthroplasty,* STD* Standardabweichung

## Footnotes

1 Da die realen Kosten für die Krankenkassen und die Kliniken nicht zu ermitteln sind, werden in dieser Arbeit die Erlöse der Kliniken pro Fall – entsprechend den von den Kassen geleisteten Vergütungen – als Kosten aufgeführt [7].

## References

[1] Becker C, Auer R, Rapp K, Jacobs K, Kuhlmey A, Greß S, Klauber J, Schwinger A (2020). Geriatrische Rehabilitation – Aktueller Stand und zukünftige Entwicklung. Pflege-Report 2020: Neuausrichtung von Versorgung und Finanzierung.

[2] Beivers A, Emde A, Klauber J, Geraedts M, Friedrich J, Wasem J, Beivers A (2020). DRG-Einführung in Deutschland: Anspruch, Wirklichkeit und Anpassungsbedarf aus gesundheitsökonomischer Sicht. Krankenhaus-Report 2020: Finanzierung und Vergütung am Scheideweg.

[3] Charlson ME, Pompei P, Ales KL (1987). A new method of classifying prognostic comorbidity in longitudinal studies: development and validation. J Chronic Dis.

[4] Court-Brown CM, Caesar B (2006). Epidemiology of adult fractures: a review. Injury.

[5] Curtis EM, Moon RJ, Harvey NC (2017). The impact of fragility fracture and approaches to osteoporosis risk assessment worldwide. Bone.

[6] Fraser AN, Bjørdal J, Wagle TM (2020). Reverse shoulder arthroplasty is superior to plate fixation at 2 years for displaced proximal humeral fractures in the elderly: a multicenter randomized controlled trial. J Bone Joint Surg Am.

[7] Frerichmann U, Raschke MJ, Stockle U (2007). Proximal femoral fractures in the elderly. Data from health insurance providers on more than 23 million insured persons—part 2. Unfallchirurg.

[8] Gödecker-Geenen N (2000). Krankenhäuser: Sozialarbeit unverzichtbar. Dtsch Arztebl.

[9] Giannotti S, Bottai V, Dell’osso G (2012). Indices of risk assessment of fracture of the proximal humerus. Clin Cases Miner Bone Metab.

[10] Giardella A, Ascione F, Mocchi M (2017). Reverse total shoulder versus angular stable plate treatment for proximal humeral fractures in over 65 years old patients. Muscles Ligaments Tendons J.

[11] Gupta AK, Harris JD, Erickson BJ (2015). Surgical management of complex proximal humerus fractures—a systematic review of 92 studies including 4500 patients. J Orthop Trauma.

[12] Haasters F, Siebenbürger G, Helfen T (2016). Complications of locked plating for proximal humeral fractures-are we getting any better?. J Shoulder Elbow Surg.

[13] Kelly BJ, Myeroff CM (2020). Reverse shoulder arthroplasty for proximal humerus fracture. Curr Rev Musculoskelet Med.

[14] King JJ, Patrick MR, Struk AM (2017). Perioperative factors affecting the length of hospitalization after shoulder arthroplasty. J Am Acad Orthop Surg Glob Res Rev.

[15] Klug A, Gramlich Y, Wincheringer D (2019). Trends in surgical management of proximal humeral fractures in adults: a nationwide study of records in Germany from 2007 to 2016. Arch Orthop Trauma Surg.

[16] Klug A, Harth J, Hoffmann R (2020). Surgical treatment of complex proximal humeral fractures in elderly patients: a matched-pair analysis of angular-stable plating vs. reverse shoulder arthroplasty. J Shoulder Elbow Surg.

[17] Knobe M, Böttcher B, Coburn M (2019). Geriatric Trauma Center DGU(R): evaluation of clinical and economic parameters: a pilot study in a german university hospital. Unfallchirurg.

[18] Köppe J, Stolberg-Stolberg J, Rischen R (2021). In-hospital complications are more likely to occur after reverse shoulder arthroplasty than after locked plating for proximal humeral fractures. Clin Orthop Relat Res.

[19] Launonen AP, Lepola V, Saranko A (2015). Epidemiology of proximal humerus fractures. Arch Osteoporos.

[20] Maugendre E, Gadisseux B, Chantelot C (2019). Epidemiology and mortality in older patients treated by reverse shoulder arthroplasty for displaced proximal humerus fractures. Orthop Traumatol Surg Res.

[21] McMillan TE, Johnstone AJ (2018). Primary screw perforation or subsequent screw cut-out following proximal humerus fracture fixation using locking plates: a review of causative factors and proposed solutions. Int Orthop.

[22] Menendez ME, Lawler SM, Carducci MP (2019). Delayed hospital discharge after total shoulder arthroplasty: why, and who is at risk?. JSES Open Access.

[23] Quan H, Li B, Couris CM (2011). Updating and validating the Charlson comorbidity index and score for risk adjustment in hospital discharge abstracts using data from 6 countries. Am J Epidemiol.

[24] Schray D, Stumpf U, Kammerlander C (2016). Diagnosis and therapy of osteoporosis in geriatric trauma patients: an update. Innov Surg Sci.

[25] Schwermann T, Grotz M, Blanke M (2004). Evaluation der Kosten von polytraumatisierten Patienten insbesondere aus der Perspektive des Krankenhauses. Unfallchirurg.

[26] Shah SS, Gaal BT, Roche AM (2020). The modern reverse shoulder arthroplasty and an updated systematic review for each complication: part I. JSES Int.

[27] Shah SS, Roche AM, Sullivan SW (2021). The modern reverse shoulder arthroplasty and an updated systematic review for each complication: part II. JSES Int.

[28] Siebert HR, Beck A (2005). Trauma surgery in the elderly. Chirurg.

[29] Soles GL, Tornetta P (2011). Multiple trauma in the elderly: new management perspectives. J Orthop Trauma.

[30] Stausberg J, Hagn S (2015). New morbidity and comorbidity scores based on the structure of the ICD-10. PLoS ONE.

[31] Stolberg-Stolberg J, Milstrey A, Schliemann B (2021). Competence, creativity and communication: basics for quality improvement in traumatology: reality and future challenges. Chirurg.

[32] Stone MA, Namdari S (2019). Surgical considerations in the treatment of osteoporotic proximal humerus fractures. Orthop Clin North Am.

[33] Tansey RJ, Almustafa M, Hammerbeck H (2020). Reverse shoulder replacement: a day-case procedure. JSES Int.

[34] Wacker F, Zapp W, Terbeck J (2014). Grundlagen der Erlösverteilung im Krankenhaus. Kosten- versus Erlösverteilung im DRG-System: Analyse – Verfahren – Praxisbeispiele.

[35] Wahnert D, Roos A, Glasbrenner J (2017). Traumatology in the elderly: multimodal prevention of delirium and use of augmentation techniques. Chirurg.

